# Is routine screening for substance use disorders useful in palliative care? Results of a prospective observational study

**DOI:** 10.1186/s13011-026-00748-z

**Published:** 2026-07-15

**Authors:** Christian Volberg, Katharina Toussaint, Jorge Riera-Knorrenschild, Felix Carl Fabian Schmitt, Astrid Morin, Martin Gschnell, Nils Heuser

**Affiliations:** 1 Marburg University, Department of Anesthesiology and Intensive Care Medicine, University Hospital of Marburg, Baldingerstraße, 35043 Marburg, Germany; 2 Marburg University, Department of Haematology, Oncology and Immunology, University Hospital of Marburg, Marburg, Germany; 3 Marburg University, Department of Dermatology and Allergology, University Hospital of Marburg, Marburg, Germany

## Abstract

**Background:**

Substance use disorders are an increasingly prevalent issue in today’s society, and they can be caused by prescription of drugs with addictive potential. Patients with incurable diseases often experience severe symptoms and require medications with the potential to cause dependence. For patients with a predisposition to addiction, this can lead to the development of a substance use disorder, further reducing their quality of life.

**Methodology:**

Over a period of six months, all patients newly admitted to the palliative care unit were screened for unusual medication use. If the initial survey, which used the ‘Short Questionnaire on Medication Use’, revealed abnormal results, the WHO-ASSIST V3.0 questionnaire was administered to identify substance use disorders and the extent of dependence.

**Results:**

A total of 181 patients were admitted to the palliative care unit during the survey period. Of these, the initial screening could not be performed in 119 patients (65.7%) due to accompanying circumstances. Of the 62 patients who were included in the study, 15 (24.2%) had an elevated score on the ‘Short Questionnaire on Medication Use’. The WHO-ASSIST V3.0 questionnaire indicated positive consumption of alcohol in 100% of cases, opioids in 86.7% of cases, tobacco in 73.3% of cases, and sedatives and sleeping pills in 53.3% of cases. Most respondents were at moderate risk of dependence, with only one person at high risk of opioid use. However, further treatment for a substance use disorder was not deemed to be clinically relevant for any of the patients.

**Conclusion:**

This survey found that routinely screening palliative care patients for substance use disorders did not provide any clinically relevant information for further treatment of the examined cohort. Given the high level of stress often experienced by people with incurable diseases, the extent to which screening is helpful or merely an unnecessary burden should be considered. As other surveys have shown that palliative care patients with substance use disorders benefit from treatment, follow-up studies should investigate how to identify patients at risk specifically.

## Introduction

Addiction is a major societal problem, directly affecting the quality of life of those affected and placing a heavy burden on their families. There are many different types of addiction, with a fundamental distinction being made between substance use disorders and behavioral addictions. The most common forms of substance use disorders involve the dependence on legal substances (e.g. tobacco or alcohol), illegal substances (e.g. heroin or cocaine), or medications with addictive potential [1]. Notably, the iatrogenic induced misuse of medications poses a significant treatment challenge that demands special attention. The opioid crisis in the United States of America is a striking example of the serious consequences that the uncontrolled prescription of medication can have [2].

Modern oncological treatment methods are enabling patients to survive for much longer than would have been possible a decade ago [3, 4]. While this is a very encouraging trend, it does mean that the prescription of drugs for symptom control, especially in palliative care, needs to be reconsidered. As palliative care patients often experience severely debilitating symptoms, they often require medications with the potential to cause dependence [5]. Opioids and benzodiazepines, in particular, are commonly used to alleviate symptoms such as dyspnoea, pain, anxiety and tension [6, 7]. These drugs have been used for many years and are an integral part of modern palliative care [8].

Substance use disorders usually develop as a result of multiple factors. The following determinants must be considered: individual factors (e.g. personality and genetic traits); environmental factors (e.g. social context); substance factors (e.g. mode of action and availability); psychological factors (e.g. lack of social skills and the addictive substance’s role in coping with this lack); and psychiatric factors (e.g. anxiety and depression). These biopsychosocial causes are maintained by the effects of conditioning, such as positive reinforcement through dopaminergic reward and negative reinforcement through the avoidance of withdrawal symptoms [1]. Palliative patients are at risk of developing substance misuse induced by treatment with opioids or benzodiazepines if they initially have a high symptom burden, but the underlying disease improves and the symptoms become less severe over time. If the medication is not reduced accordingly, and if access to non-retard medication is still available, patients with a predisposition to this may become dependent on the substances due to their inappropriate use. Patients with a lack of social support, poor coping skills, and accompanying anxiety or depression are particularly at risk. Previous episodes of substance misuse can also contribute to the development of a new addiction [9–11].

## Methodology

This prospective cross-sectional study was submitted to the Ethics Committee of the Faculty of Medicine at Philipps University of Marburg for review and received approval on 27 August 2024 (24–241 ANZ). Data were collected between 1 October 2024 and 31 March 2025 in the palliative care unit at Marburg University Hospital in Germany. All patients admitted to the palliative care unit during the survey period underwent routine clinical screening for inappropriate medication use. This was done using the Short Questionnaire on Medication Use by Watzl et al., comprising eleven questions on medication use requiring a dichotomous yes/no answer [12]. If the screening yielded a total score of at least four positive answers, a more detailed screening was carried out using the German version of the WHO-ASSIST V3.0 questionnaire (Alcohol, Smoking & Substance Involvement Screening Test) [13, 14]. The WHO-ASSIST V3.0 is a self-reporting tool that helps to identify substance use disorders and the degree of dependence. It asks about various substances (tobacco, alcohol, cannabis, cocaine, amphetamines, sedatives, hallucinogens, inhalants, opioids, and other substances), how often they are used, whether family or friends are concerned about their use, and whether the patient has tried to reduce the use [14].

If the questionnaire yielded a positive result for a substance use disorder, further treatment options were discussed with the patient, and if necessary, a targeted treatment plan was drawn up. If a patient was readmitted to the palliative care unit during the survey period, no further screening was carried out.

Patients were excluded if they were unable to participate in the survey, had language barriers, cognitive impairments or being in the terminal phase. For patients who were unable to participate, only demographic data and the reason for non-participation were recorded for statistical analysis.

The anonymised data were transferred to IBM Statistics Version 30.0.0.0 (172) for initial descriptive evaluation. Descriptive measures such as the mean, standard deviation, minimum and maximum, and the associated confidence intervals were calculated. For non-normally distributed variables, the corresponding non-parametric measures of location (median and interquartile range) were calculated. Frequency differences were tested for significance using a chi-squared four-field test (Fisher’s exact test was used for small sample sizes). Dichotomous distributions were tested for significance using the point-biserial correlation coefficient. The significance level was set at α = 0.05.

## Results

**Fig. 1:**
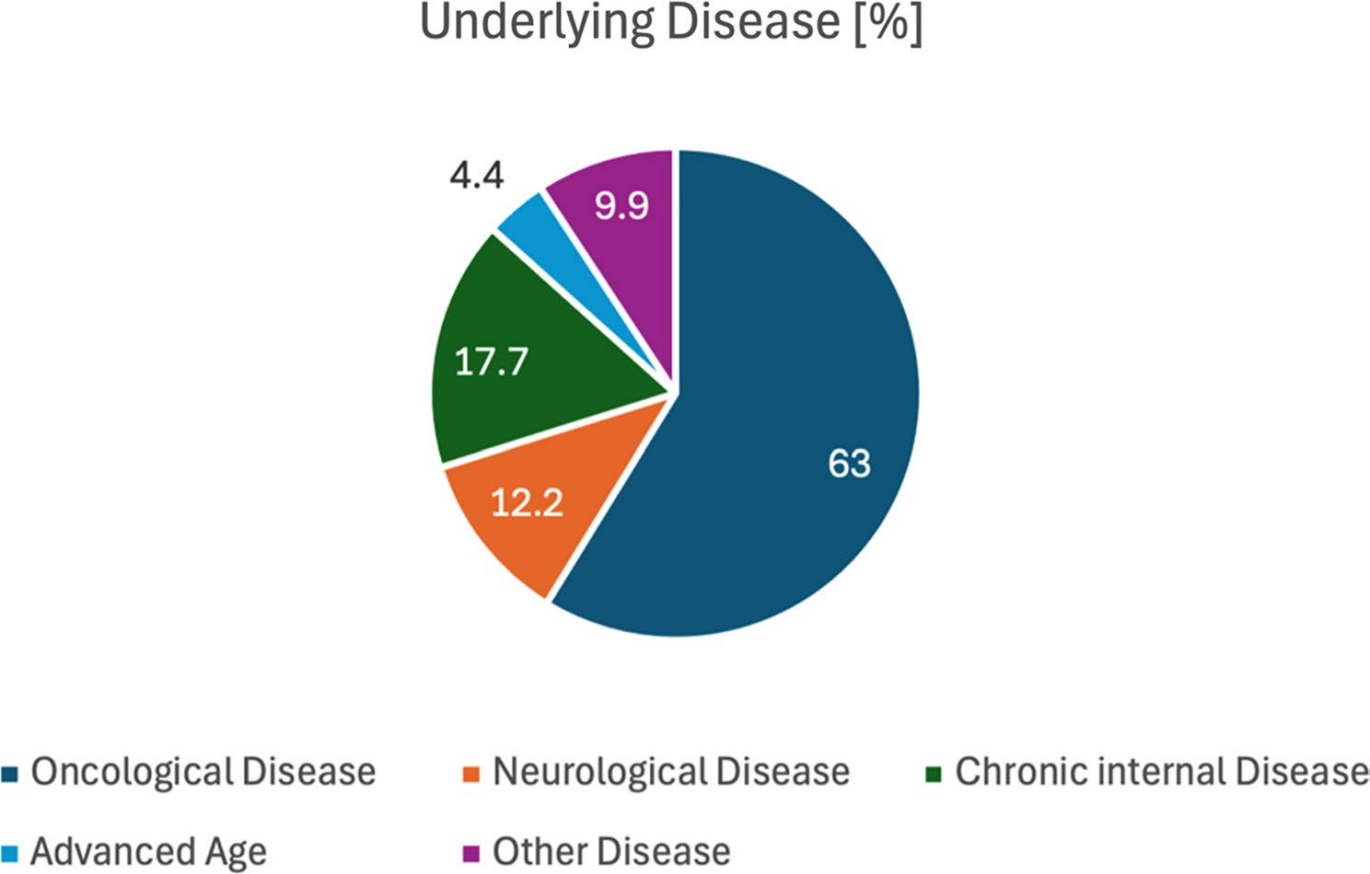
Underlying disease of patients

During the six-month survey period, a total of 181 patients were admitted to the palliative care unit for the first time and screened for substance use disorders. The entire cohort comprised almost equal numbers of men and women, with an average age of 74.2 ± 13.3 years. The majority of patients (63%) suffered from an underlying oncological disease (see Fig. 1).

A total of 119 patients (65.7%) could not be interviewed. Excluded patients were significantly older than included patients and were more frequently admitted to the palliative care unit due to chronic internal diseases and neurological diseases (see Table 1). The most common reason for being unable to conduct the survey was that patients were dying (46.2%), too weak (24.5%), or confused (18.5%) (see Table 2).

**Table 1:** Comparison of the demographics of included and excluded patients. Multiple responses were possible when specifying the underlying disease

	Inclusion	Exclusion	*p*
**Number of patients [%]**	62 (34.3%)	119 (65.7%)	
**Age [y]**	66.5 ± 12.8	78.7 ± 11.7	< 0.001
**Gender [n]**			
Male	30 (48.4%)	61 (51.3%)	0.71
Female	32 (51.6%)	58 (48.7%)	
**Underlying disease [n]**			
Oncological	56 (90.3%)	49 (41.2%)	< 0.001
Chronic internal disease	4 (6.5%)	26 (21.8%)	0.08
Neurological	1 (1.6%)	19 (16%)	0.02
Advanced age	0 (0%)	6 (5%)	0.1
Other	2 (3.2%)	15 (12.6%)	0.06

**Table 2:** Reasons for exclusion from the survey

Total	119 (65.7%)
Dying	55 (46.2%)
Weakness	29 (24.5%)
Confusion/Delirium	22 (18.5%)
Dementia	8 (6.7%)
Coma	5 (4.2%)
Stress	4 (3.4%)
Refused to be interviewed	2 (1.7%)
Other reason	17 (14.3%)

A total of 62 patients took part in the survey, completing the Short Questionnaire on Medication Use. Their responses are shown in Table 3. Notably, patients frequently stated that they cannot fall asleep without their medication (43.8%), and that they want to temporarily take a break from everything (41%). Medication for pain relief was also frequently taken (39.7%).

**Table 3:** Responses to the short questionnaire on medication use (*n* = 62)

Statement	Yes [*n*]	No [*n*]
I cannot fall asleep without medication.	26 (41.9%)	36 (58.1%)
I have already stocked up on a small supply of tablets, just to be on the safe side.	11 (17.7%)	51 (82.3%)
Sometimes I want to withdraw from everything.	25 (40.3%)	37 (59.7%)
There are situations that I cannot cope with without medication.	16 (25.8%)	46 (74.2%)
Others believe that I have problems with medication.	3 (4.8%)	59 (95.2%)
My medication is no longer as effective as it was at the beginning.	16 (25.8%)	46 (74.2%)
I often take medication because I am in pain.	25 (40.3%)	37 (59.7%)
When I take more medication, I eat less.	10 (16.1%)	52 (83.9%)
I don’t feel well without medication.	18 (29%)	44 (71%)
Sometimes I was surprised how many tablets I took in a day.	19 (10.6)	43 (69.4%)
I often feel more productive when I take medication.	14 (22.6%)	48 (77.4%)

Fifteen patients (24.2%) had a positive score on the Short Questionnaire on Medication Use (total score of ≥ 4 positively answered questions), so a more specific questionnaire was conducted using the WHO-ASSIST V3.0. This revealed that all respondents had consumed alcohol at some point in their lives and that a large proportion (73.3%) had consumed tobacco. Similarly, most of them (86.7%) had taken opioids, and over half (53.3%) had taken sedatives or sleeping pills. Only a few had consumed illegal drugs (cocaine and amphetamines) (see Table 4). The risk of substance misuse was moderate for 92.3% of patients taking opioids, and high for one person. Moderate risk was also found for almost all individuals who had taken sedatives and sleeping pills, as well as for tobacco consumption.

**Table 4:** Results of the WHO-ASSIST V3.0 questionnaire

Substance	Consumption	Risk: low	Risk: medium	Risk: high
Tobacco	11 (73.3%)	4 (36.4%)	7 (63.6%)	0 (0%)
Alcohol	15 (100%)	13 (86.7%)	2 (13.3%)	0 (0%)
Cannabis	2 (13.3%)	1 (50%)	1 (50%)	0 (0%)
Cocaine	1 (6.7%)	1 (100%)	0 (0%)	0 (0%)
Amphetamines	1 (6.7%)	1 (100%)	0 (0%)	0 (0%)
Inhalants	0 (0%)	0 (0%)	0 (0%)	0 (0%)
Sedatives and sleeping pills	8 (53.3%)	1 (12.5%)	7 (87.5%)	0 (0%)
Hallucinogens	0 (0%)	0 (0%)	0 (0%)	0 (0%)
Opioids	13 (86.7%)	0 (0%)	12 (92.3%)	1 (7.7%)
Other	0 (0%)	0 (0%)	0 (0%)	0 (0%)

A striking finding when comparing the data from the WHO-ASSIST V3.0 questionnaire with the patient records is that all patients were already taking opioids as part of their long-term medication when admitted to the palliative care unit. Similarly, five patients had been prescribed a cannabis preparation and five patients also benzodiazepines (compare Tables 4 and 5).

**Table 5:** The prescribed medications of the 15 patients who achieved elevated screening values and completed the WHO-ASSIST V3.0 questionnaire upon admission to the palliative care unit

Medication	Already prescribed upon admission
Fentanyl	6 (40%)
Oxycodone	4 (26.7%)
Hydromorphone	3 (20%)
Morphine	2 (13.3%)
Cannabis (e.g. Dronabinol)	5 (33.3%)
Lorazepam	5 (33.3%)
Pregabalin	8 (53.3%)

## Discussion

When providing palliative care to patients with substance use disorders, a distinction must be made between two fundamentally different scenarios. On the one hand, patients with long-standing addiction disorders, or those who have overcome them, may find themselves in a palliative care situation. On the other hand, patients with incurable diseases may develop an iatrogenic induced substance use disorder [15]. The first group is at risk of undersupply due to an increased need for analgesics or sedatives, and practitioners’ reluctance to prescribe such large quantities of medication. Furthermore, abstinent patients may relapse into substance use disorder. Iatrogenic induced substance use disorder is particularly likely when patients are predisposed to addiction, when improper prescribing creates free access to addictive substances, and when patients develop a dependence on these substances [15]. Both scenarios present challenges for doctors and nursing staff when it comes to treating patients with substance use disorders. Although palliative care physicians are well trained in symptom control, they may not have experience of treating patients with addictions [16]. Initial projects are emerging, such as at the University of Texas, which aim to provide adequate treatment for patients with incurable underlying diseases and accompanying substance use disorders [17, 18]. In this context, the importance of meaningful cooperation between palliative care physicians and addiction experts is emphasised [15]. Improper opioid use can lead to severe side effects in the final stages of life, including constipation, delirium and, in the worst cases, respiratory depression, all of which should be avoided [19]. A systematic review by Robyn Keall et al. found that young age, a personal or family history of mental illness, and a history of illegal drug use in particular are associated with an increased risk of addiction in palliative care patients [20]. These findings are also supported by a study conducted by Marti et al. in Switzerland. In their retrospective study, they demonstrated that patients with substance use disorders were predominantly single, childless, living alone, and either unemployed or receiving a disability pension. Psychiatric and infectious diseases were often comorbid, and those affected by a substance use disorder were significantly younger than the comparison group, significantly more likely to be male, and reported more severe pain [21].

The data from our study show that, while general screening of patients in a palliative care unit is possible, it also has limitations that must be taken into account. Firstly, a large proportion of patients (65.7%) were unable to participate in the screening due to their general condition. This finding is consistent with the results of other studies that have shown that most patients admitted to a palliative care unit are in poor health [22]. Of those who could be surveyed in our cohort, 24.2% had an elevated initial screening score. This figure is consistent with that found by Pooja Kumar and her colleagues when they screened patients with advanced lung cancer for substance use disorders. In their study, 30.8% of patients scored positively in the questionnaire screening [23]. For our patients, scoring 4 or more positive answers in the general screening led to a more detailed assessment of substance use using the WHO-ASSIST V3.0 questionnaire. The results for alcohol, opioids, tobacco, and sedatives were particularly positive. However, most individuals showed a low to moderate risk of developing a substance use disorder. Alcohol and tobacco generally lead to increased dependence in society due to their legal availability [24]. A comparison of the medication plans of the surveyed patients revealed that all of them had been prescribed opioids. The WHO-ASSIST V3.0 questionnaire was designed solely to identify excessive medication use, not to evaluate prescriptions issued by physicians. Therefore, the results suggest that a certain proportion of patients with a valid prescription are not taking their medication as directed by their doctor. It can be assumed that many patients are unaware of the medications they are taking, including the exact indications for their on-demand medications. Heuser et al. have highlighted this issue and demonstrated that patients frequently lack knowledge about their tumour stage and the specific therapy they are receiving [25]. Not knowing about one’s own treatment can therefore be dangerous, particularly when potent drugs such as opioids or sedatives are taken without individuals knowing how they work or what side effects they may experience. None of the patients treated during the study period showed signs of alcohol or drug misuse in their clinical course. This demonstrates that substance use disorder was very rare among the patients in our study. Therefore, an increased prevalence due to positive test results (e.g. from urine tests), as observed in other studies, cannot be directly applied to German palliative care patients. These studies were mostly conducted in the United States of America in the context of the opioid crisis there. Despite the higher prevalence, the general use of urine drug screening in palliative care patients is a controversial topic in the literature [20, 26].

However, an increasing amount of international literature points to a conflict between, on the one hand, patients who are not receiving adequate pain treatment and, on the other hand, the high level of opioid dependence among patients. These controversial perspectives, known as the ‘pain pandemic’ and the ‘opioid addiction epidemic’, hinder the delivery of effective care. Fear of addiction can result in insufficient analgesics being prescribed, or patients refusing to take them. Conversely, inappropriate prescribing can also lead to addiction in vulnerable individuals [27]. Structured screenings are rarely carried out routinely in Europe [28]. Surveys conducted by Eersink et al. among palliative care providers in Germany also showed that screenings on palliative care units and in specialised outpatient palliative care services are seldom carried out. Instead, palliative care physicians who participated in the surveys often stated that they were more concerned about inadequate pain management than substance dependence in patients with low life expectancy [29, 30]. The evaluation by Klint et al. from Sweden also showed that pain management is often inadequate, as patients suffered from poorly controlled pain in the last weeks of their lives despite being prescribed opioids [31]. The negative effects of the opioid crisis in the United States also influence other countries, although this phenomenon cannot easily be transferred. Strict legal regulations on opioid prescriptions, such as those in place in Germany, prevent inappropriate prescribing and counteract the procurement of potentially addictive drugs [27].

## Limitations

One limitation is the single centre design of the study at one German University Hospital. Comparing results from different institutions and care areas (rural versus urban) would provide a more comprehensive picture. Due to the small number of cases and the high proportion of patients unable to participate in the survey, the results’ significance is limited.

The WHO-ASSIST V3.0 questionnaire was developed to record substance use and its negative effects [14]. It can be assumed that the surveyed patients understood the instructions for completing the questionnaire correctly and did not misreport their medication adherence. However, it is not possible to completely rule out false statements.

Nevertheless, this study provides initial findings which show that substance abuse currently plays a minor role in palliative care.

## Conclusion and outlook

As patients in palliative care are often under considerable stress, unnecessary examinations and diagnostics should be avoided unless they benefit the patient. This aspect must be assessed on an individual basis.

According to the available data, general screening for substance use disorders in palliative care patients is not recommended. However, raising awareness of the issue is important in order to identify patients at risk or who may already have a problem, and to offer them expert help. In this context, it is important to consider the extent to which the addiction is impairing the patient’s quality of life, how much rest lifetime has left, and whether the patient wants to undergo withdrawal treatment.

Current screening questionnaires do not address the specific requirements of palliative care patients, who frequently receive potentially addictive medications for symptom management. It is therefore necessary to develop screening tools that are specifically tailored to the needs of palliative care patients.

## Data Availability

The datasets used and analyzed in the current study are available from the corresponding author on reasonable request.
